# The Intensity of Early Attentional Processing, but Not Conflict Monitoring, Determines the Size of Subliminal Response Conflicts

**DOI:** 10.3389/fnhum.2019.00053

**Published:** 2019-02-20

**Authors:** Wiebke Bensmann, Amirali Vahid, Christian Beste, Ann-Kathrin Stock

**Affiliations:** Cognitive Neurophysiology, Department of Child and Adolescent Psychiatry, Faculty of Medicine Carl Gustav Carus, TU Dresden, Dresden, Germany

**Keywords:** attention, frontoparietal network, machine learning, subliminal priming, task set

## Abstract

Response conflicts hamper goal-directed behavior and may be evoked by both consciously and subliminally (unconsciously) processed information. Yet, not much is known about the mechanisms and brain regions driving the size of subliminally induced conflicts. We hence combined a response conflict paradigm featuring subliminal primes and conscious flankers with in-depth neurophysiological (EEG) analyses, including source localization in a sample of *N* = 243 healthy subjects. Intra-individual differences in the size of subliminal conflicts were reflected both during early attentional stimulus processing (prime-associated N1 and target-associated P1 and N1 amplitudes) and conflict monitoring (N2 amplitudes). On the neuroanatomical level, this was reflected by activity modulations in the TPJ (BA39, BA40) and V2 (BA18), which are known to be involved in attentional stimulus processing and task set maintenance. In addition to a “standard” analysis of event-related potentials, we also conducted a purely data-driven machine learning approach using support vector machines (SVM) in order to identify neurophysiological features which do not only reflect the size of subliminal conflict, but actually allow to classify/predict it. This showed that only extremely early information processing (about 65 ms after the onset of the prime) was predictive of subliminal conflict size. Importantly, this predictive feature occurred before target information could even be processed and was reflected by activity in the left middle frontal gyrus (BA6) and insula (BA13). We conclude that differences in task set maintenance and potentially also in subliminal attentional processing of task-relevant features, but not conflict monitoring, determine the size of subliminally induced response conflicts.

## Introduction

Exerting cognitive control over one’s actions is expedient to living a self-serving and successful life. An important aspect of cognitive control is the ability to select a required response among competing alternatives. Yet, response selection is known to be quite error-prone, as processes required for the correct response can often not be sufficiently shielded from irrelevant information and/or competing (incorrect) response tendencies (Goschke and Dreisbach, 2008; Keye et al., 2013; Beste et al., 2017; Stock et al., 2017b). Such response conflicts increase error rates and delay correct responses (Goschke and Dreisbach, 2008), so that the size of a response conflict is usually quantified by assessing how much the behavioral performance worsens when a conflict is induced.

Importantly, response selection may not only be hampered by consciously perceived input, but also by subliminally perceived information (Eimer and Schlaghecken, 2003; Schlaghecken and Eimer, 2004; McBride et al., 2012; Parkinson and Haggard, 2014; Ulrich et al., 2015; Gohil et al., 2017). Subliminal and consciously perceived distractors are known to evoke different kinds of response conflicts (e.g., Boy et al., 2010b; Stock et al., 2016a; Bensmann et al., 2018). These two kinds of conflicts differ from each other as consciously perceived distractors/conflicts may trigger consciously initiated top-down control processes (Dehaene and Naccache, 2001; Kiefer et al., 2011; Stock et al., 2016a) and modulate brain activity in “classical” conflict monitoring regions such as the anterior cingulate cortex (ACC) (Desender and Van den Bussche, 2012) while this does not seem to hold true for subliminally perceived conflicts (Dehaene et al., 2003; Boy et al., 2010b). Overall, the modulation of conflict monitoring, effort and response selection has been rather well investigated in consciously triggered response conflicts (Botvinick et al., 2004; Botvinick, 2007; Chmielewski et al., 2014; Larson et al., 2014; Mückschel and Beste, 2015; Stock et al., 2016a), but only little is known about how subliminally triggered response conflicts arise and which factors determine their size (Desender and Van den Bussche, 2012; Huber-Huber and Ansorge, 2017, 2018). It has, however, already been shown even that conflict awareness is neither required for subliminal priming, nor for and conflict sequence effects thereon (Huber-Huber and Ansorge, 2017, 2018).

In the recent past, several authors have started to study subliminal priming in the context of supraliminal response conflicts to investigate how the two kinds of conflict interact (e.g., Boy et al., 2010b; Stock et al., 2016a; Gohil et al., 2017; Bensmann et al., 2018). However, the results were far from straightforward, showing non-additive influences of subliminal and supraliminal conflict. In our view, a complex experimental condition, such as the interaction of supra- and subliminal conflict, cannot be understood by using behavioral outcomes alone. Instead, this situation requires further physiological measures and invites less assuming approaches that are more open to unanticipated results, such as applying relatively unassuming machine learning to the temporally highly resolved human electroencephalogram (EEG). In the current study, we took this approach with a focus on the less well-researched, subliminally induced response conflicts and how they relate to consciously perceived conflicts.

In order to investigate potential determinants of subliminal response conflict size (and its potential interaction with conscious conflicts), we applied a paradigm that allows to investigate both consciously and subliminally induced response conflicts by combining response-relevant targets with two different kinds of distractors (i.e., subliminal primes and consciously perceived flankers) (Stock et al., 2016a). In order to assess subliminal response conflicts, we quantified the positive compatibility effect (PCE), which is characterized by faster responses in case the primed automatic response tendency is compatible with the required response to the target (Eimer and Schlaghecken, 2003). We then chose to contrast subjects with large and small PCEs as this allows to investigate which factors determine the size of a subliminal response conflict (i.e., the PCE).

Importantly, taking a relatively unassuming machine learning approach does, however, not mean that we had no hypotheses at all. Behavioral hypotheses may be deduced from findings and models about how subliminal information modulates behavior in general. It has been suggested that “instructed facts would be organized into a task set; a temporary coding scheme that proactively tunes sensorimotor pathways according to instructions to enable highly efficient ‘reflex-like’ performance” (Muhle-Karbe et al., 2017; see also Neumann and Klotz, 1994; Kunde et al., 2003; Ansorge and Neumann, 2005). Active task sets are commonly thought to be represented in frontoparietal areas and to regulate the responsiveness to task-relevant stimulus features in both primary sensory areas and sensory association cortices (Kiefer, 2008; Kiefer et al., 2011; Muhle-Karbe et al., 2017). Based on this mechanism and because processing of task-relevant features does not necessarily require any form of conscious processing (van Boxtel et al., 2010; van Gaal et al., 2012; Muhle-Karbe et al., 2017), subliminal priming may bias early information accumulation and activation in decision circuits (Leuthold and Kopp, 1998; Vorberg et al., 2003; Parkinson and Haggard, 2014), which ultimately results in an automatic response tendency (Eimer and Schlaghecken, 1998, 2003; Eimer, 1999; Schlaghecken and Eimer, 2000). Depending on whether this automatic response tendency converges or conflicts with a consciously initiated, top-down response, it may either facilitate or hamper behavioral performance, which gives evidence for a subliminal priming effect (Eimer and Schlaghecken, 1998, 2003; Eimer, 1999; Schlaghecken and Eimer, 2000). It hence seems likely that the size of subliminally triggered response conflicts might be determined by differences in the strength of task set representations, which likely determine how much task-irrelevant information is attended, processed and subsequently converted into automatic response tendencies. This implies that the size of (subliminally) triggered conflicts might be determined by differences in the efficiency of task set representations and/or early attentional stimulus processing, rather than by differences in conflict monitoring or response selection processes.

In order to dissociate different cognitive sub-processes, we recorded an EEG during task performance, as ERPs are well-suited to distinguish attentional and control-related processes. Since it has been suggested that subliminal priming may bias early information accumulation (Scharlau and Ansorge, 2003), we assessed early attentional stimulus processing by quantifying the prime- and target-associated P1 and N1 components (Luck et al., 2000). For ERP quantification and labeling, we determined the association with prime or target on the basis of temporal proximity. This means that prime-associated components occur shortly after the onset of the prime, while target-associated components occur shortly after the onset of the target. It should, however, be noted that despite this labeling, especially the latter ones may reflect aspects of both prime and target processing. Importantly, researchers found that without attention being successfully directed to the target and, hence, also to the subliminal primes, congruence effects based on prime-target motor conflict were much weaker (Naccache et al., 2002). This nicely matches our initial hypothesis, that the size of subliminal conflicts may be driven by the intensity of initial stimulus processing. Therefore, we expected larger prime-associated P1 and N1 amplitudes in individuals with larger subliminal conflict/PCEs. Given that conflicting stimulus input may increase P1 and N1 amplitudes (Ernst et al., 2013), we further expected larger target-associated P1 and N1 amplitudes whenever primes and/or flankers are not compatible with the target. As already stated above, this should be mostly reflected in attentional networks (Vossel et al., 2014). Yet, cognitive conflicts and associated control mechanisms are still often assumed to mainly unfold during later processing stages (Botvinick et al., 2001; Bocanegra and Hommel, 2014; Ulrich et al., 2015; Stock et al., 2017a) We hence also quantified the N2 component, which is known to increase in case of conflicts (Botvinick et al., 2004; Folstein and Van Petten, 2007; Willemssen et al., 2009; Beste et al., 2010; Chmielewski et al., 2014; Petruo et al., 2016). Based on this, we expected the modulation of N2 amplitudes to reflect conflict size, with larger amplitude (increases) being related to larger subliminal conflicts/PCEs as well as conscious (flanker) conflicts (i.e., N2 amplitudes should be smallest in case of compatible primes paired with congruent flankers, larger in case of either incompatible primes or incongruent flankers and the largest in case of incompatible primes paired with incongruent flankers). Lastly, we quantified the parietal P3 amplitude, which is commonly thought to reflect stimulus–response mapping (Verleger et al., 2005, 2015). Given that prepared reflexes are thought to be driven by stimulus–response associations (Hommel, 2009; Muhle-Karbe et al., 2017), we expected to find larger P3 amplitudes in individuals with larger PCEs and/or flanker effects.

With respect to brain regions may be functionally associated with the expected effects and may be identified from the EEG signal, we expected to find differences in frontoparietal networks. The reasoning behind this assumption is that task set representation has most commonly been attributed to frontoparietal networks, including the lateral prefrontal cortex (PFC) and middle frontal gyrus (MFG) and/or the parietal cortex including the temporo-parietal junction (TPJ) (Crone et al., 2006; Bengtsson et al., 2009; Kiefer et al., 2011; Loose et al., 2017; Muhle-Karbe et al., 2017). Regions that reflect differences in attentional processing (of subliminal stimuli) partly overlap with these networks and comprise visual and frontoparietal areas including the TPJ and supplementary motor area (SMA) (Naccache and Dehaene, 2001; Boy et al., 2010a; D’Ostilio et al., 2012; Vossel et al., 2014; Ulrich and Kiefer, 2016). As both subliminal and conscious conflicts are often detected and investigated with the N2 ERP (Larson et al., 2014), we furthermore expected to find differences in N2-associated brain regions. For the N2, it has repeatedly been demonstrated that consciously processed conflicts reliably modulate mid-frontal structures like the ACC (Botvinick et al., 1999, 2004). For subliminal primes, the picture is, however, less clear: While some EEG studies have found evidence that masked primes modulate the ACC (Desender et al., 2016), fMRI studies using masked primes do typically not find prime-associated ACC modulations (Dehaene et al., 2003). Thus, we rather expected differences in frontoparietal networks to underlie effects of subliminal priming (Desender and Van den Bussche, 2012) than the ACC.

One major strength of ERP analyses is that they are well-known to correlate with behavioral performance in a wide range of different tasks. Using ERPs, we can determine cognitive sub-processes which may contribute to task performance with a high temporal resolution and draw on a wealth of literature to interpret our findings. However, it has remained largely unclear whether classical ERPs are truly the best reflection of variations in behavior. It is even less certain that ERPs (i.e., defined minima and maxima, which result from combination of different, not necessarily synchronous source activations) can properly reflect the neuronal activity underlying behavioral variations. The reason for this is that classical ERP analyses are only correlative in nature and often limited to a small number of neurophysiologic features (i.e., a few minima and maxima at predefined electrodes and time windows), so that potentially meaningful differences in other electrodes or time domains may easily be missed. This bias is especially dramatic given that any given EEG signal is composed of different signals, which do not only vary in latency, but also stem from different neuronal generators within the human brain (Huster et al., 2013; Keil et al., 2014). In order to overcome those limitations, many recent studies have applied complex signal decomposition approaches (e.g., Brunner et al., 2013; Huster et al., 2013). Yet still, even those approaches usually limit themselves to minima and maxima of the different identified components and have likewise remained correlational. Fortunately, the recent rise in machine learning approaches and methods has equipped us with a new and powerful tool that can expediently and objectively identify differences in the entire EEG signal and may furthermore allow to identify neurophysiological features that allow to classify (“predict”) behavioral performance, instead of just correlating with it^1^. Thus, to identify the cognitive sub-processes that allow to *classify/predict* inter-individual (i.e., relative, not absolute or categorical) differences in the size of subliminally induced response conflicts, we applied a purely data-driven machine learning approach in combination with a support vector machine (SVM) on the neurophysiological data in order to complement the classical ERP quantification approach and be able to identify potential predictive features “outside” of the typically analyzed ERPs. As the data-driven feature selection draws on all available electrodes and time windows, these additional analyses gave the opportunity to test the relevance of different neurophysiological features. In combination with source localization of the obtained relevant features, it furthermore allows to identify the functional neuroanatomical structures which likely classify/predict (and not just correlate with) differences in subliminal conflict size. To investigate the potential determinants of subliminal response conflict size (and its potential interaction with conscious conflicts), we examined which features in the time domain (i.e., ERPs) best classify/predict performance/the size of subliminal response conflicts as operationalized by PCE group membership (see “Materials and Methods” section for details). In line with our main hypothesis, we expected predictive ERP features to be most likely occurring during early attentional stimulus processing, i.e., temporally close to stimulus onset. As we hypothesized that differences in subliminal conflict size should be rooted in task set representation and/or the early stimulus processing influenced thereby, we expected our predictive features to be based on activation differences in frontoparietal networks and sensory cortices (e.g., Vossel et al., 2014; Muhle-Karbe et al., 2017). Given that subliminally induced conflicts may interact with consciously perceived ones (Boy et al., 2010a; Stock et al., 2016a), we further hypothesized that inter-individual differences in the size of subliminal conflicts might modulate the size of consciously perceived ones.

In short, the main objective of this study is to identify cognitive sub-processes and associated neuroanatomical structures that allow to classify/predict inter-individual differences in the size of subliminal response conflicts, instead of just correlating with them. For this purpose, we formed performance groups based on PCE magnitude and used a data-driven machine learning approach to complement/extend regular ERP analyses. We expected to find an ERP feature reflecting task set representation and/or early attentional stimulus processing (as defined by temporal proximity to stimulus onset) to be the most likely predictor/classifier of subliminal response conflict size.

## Materials and Methods

### Participants

A group of *N* = 251 healthy young subjects participated in the study, which was part of a larger data collection, the results of which have so far not been published anywhere. The large sample was necessary because a sufficient set of training data sets is pivotal for an adequate prediction with SVM. While there are no clear-cut recommendations comparable to a regular power estimation or the like, we would strongly advise to try to collect at least 200 data sets (i.e., have at least 100 subjects per group) when trying to predict a non-clinical/behavioral group membership on the basis of EEG data.

*N* = 8 participants were excluded from analysis due to poor performance (mean accuracy ≤ 75%) or noisy EEG signal (when artifacts could not be sufficiently removed with the help of an ICA), so that data analysis was performed with *n* = 243 subjects (170 females; mean age 23.86; SD 3.20; range 18–32 years). All participants had normal or corrected-to-normal vision, and had been recruited using flyers and online ads at the local University (TU Dresden, Germany). Participants had no history of neurological or mental illness, gave written informed consent before starting the experiment and were reimbursed with 10 € after their participation. The study was approved by the ethics committee of the Faculty of Medicine of the TU Dresden and conducted in accordance with the Declaration of Helsinki.

### Task

The task was based on an experimental paradigm by Boy et al. (2010b) and identical to the paradigm used in a previous study of our group (Stock et al., 2016a). By combining the target stimulus with a subliminal prime as well as with flankers, this task allows to investigate both consciously and subliminally induced response (selection) conflicts.

**FIGURE 1:**
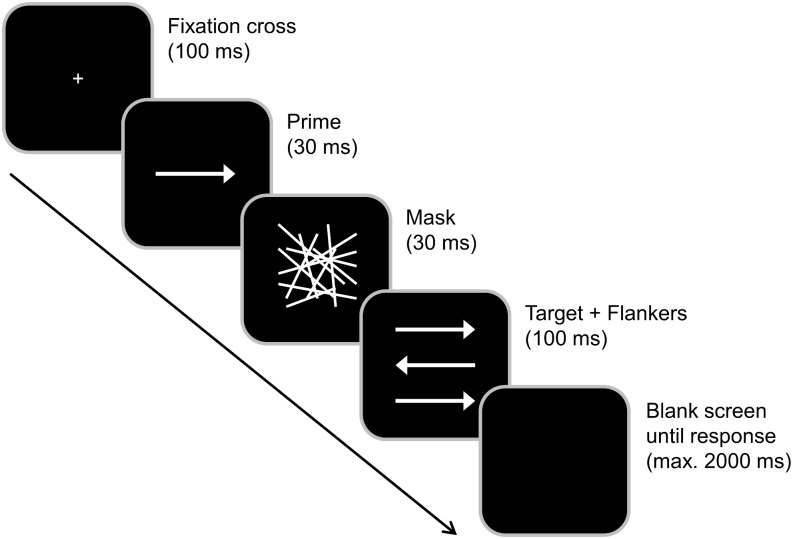
Experimental paradigm. Each trial started with a 100 ms presentation of a fixation cross, which was followed by a 30 ms presentation of a prime (middle arrow) and 30 ms presentation of a mask (array). The target (middle arrow) plus flankers were then simultaneously presented for 100 ms. After the presentation of the target, the screen turned black. Primes pointing in the same direction as the target were classified as compatible while flankers that pointed in the same direction as the target were classified as congruent.

Subjects were seated at a distance of 57 cm from a 17 inch CRT monitor and were asked to respond using the two “Ctrl” buttons on a Cherry keyboard. Participants had to rest their fingers on the response buttons during the entire experiment. “Presentation” software (Version 17.1 by Neurobehavioral Systems, Inc.) was used to present stimuli, record the behavioral responses and synchronize with the EEG. Before the start of the experiment, subjects completed a supervised task practice until they were able to comply with the task. During the practice, feedback about the accuracy of the response was provided. The experiment/data collection did not comprise response feedback. Each trial started with the central presentation of a white fixation cross on black background for 100 ms (see Figure 1). It was followed by the subliminal prime (a centrally presented horizontal white arrow pointing either to the right or left) for 30 ms, a mask (an array of randomly distributed white lines) for 30 ms and the combination of a target (a centrally presented horizontal white arrow pointing either to the right or left) and two flankers (white arrows located above and below the target) for 100 ms. All arrows had the same size. Participants were asked to focus on the target and ignore the flankers. They were instructed to indicate the pointing direction of the target arrow by pressing the right Ctrl button with the right index or middle finger in case the target arrow pointed to the right and the left Ctrl button with the left index or middle finger in case the target pointed to the left. Each trial ended with the first given response or after 2,000 ms had elapsed (in this case, the trial was coded as a “miss”). The response–stimulus interval between the participants’ response and the onset of the following trial varied randomly between 1,000 and 1,200 ms. In case the prime and target arrows pointed into the same direction, the trial was classified as compatible (and as incompatible in case of opposite pointing directions). Whenever flankers and target pointed in the same direction, trials were rated as congruent (and as incongruent in case of pointing in the other direction). Each participant completed 384 trials that were subdivided into four blocks. All possible combinations of prime compatibility, flanker congruency and target pointing direction occurred with equal frequency and their order was randomized within each block. In total, the experiment took approximately 15 min to complete. After completing the task, the participants were asked whether they had consciously perceived the prime stimulus (i.e., whether they had consciously perceived any visual stimulus preceding the mask, which we termed “scrambled lines” for the sake of better understanding). This was denied by all of them and matches the reports by Boy et al. (2010b) who reported forced choice identification rates between 46.5 and 51.9% and thus no conscious perception of the prime at a SOA of 70 ms (i.e., even 10 ms longer than in our study). Even though we did not conduct a forced choice test ourselves, we hence deem it very unlikely that participants were able to consciously perceive the trials. Prior to working on the task described in this publication, the participants spent 30 min performing another, unrelated control task (Bocanegra and Hommel, 2014; Stock et al., 2016b), the results of which have not been published so far.

### EEG Recording and Standard ERP Analysis

EEG data were recorded from 60 Ag-AgCl electrodes at standard equidistant scalp positions against a reference electrode at position Fpz using a QuickAmp amplifier (Brain Products, Inc). During recording, electrode impedances were kept below 5 kΩ, and a sampling rate of 500 Hz was employed. Brain Vision Analyzer 2.1 was used for offline data pre-processing and ERP data analyses. During this process, data were down-sampled to 256 Hz and a band-pass filter ranging from 0.5 to 20 Hz with a slope of 48 db/oct each was applied. The EEG data was average-referenced and a manual raw data inspection was used to eliminate rare technical or muscular artifacts. Subsequently, an automatic independent component analysis (ICA; infomax algorithm) was run to remove periodically recurring artifacts such as eye blinks, saccades or pulse for all participants. Lastly, another raw data inspection was conducted to remove any residual artifacts.

In the next step, EEG data were segmented in a target-locked fashion. All epochs started 2,000 ms before and ended 2,000 ms after target stimulus onset (set to time point zero). Only correctly answered trials were included in analysis. By applying an automated artifact rejection, segments with amplitudes below -100 and above 100 μV were excluded and the maximally allowed value difference in a 200 ms interval was 200 μV. Additionally, the lowest acceptable amplitude difference in a 100 ms time span was set to 0.5 μV. The reference potential was eliminated using a current source density (CSD) transformation. The CSD operates as a spatial filter and thus helps to identify the electrodes that best reflect activity related to the respective ERP (Perrin et al., 1989; Nunez and Pilgreen, 1991; Kayser and Tenke, 2015). Next, a baseline correction from -500 to -200 ms before target onset was performed (i.e., the baseline was set before the onset of the prime stimulus). Averaging of the different factor combinations/experimental conditions was separately conducted for each participant. Based on this, the P1, N1, N2, and parietal P3 ERPs were quantified. Electrodes were chosen based on visual inspection of the scalp topography, which was validated and confirmed by a procedure described in Mückschel et al. (2014): The mean amplitudes of the ERP components in the corresponding search intervals were extracted at all electrode positions at the single subject level. Subsequently, each electrode was compared to the average of all other electrodes using Bonferroni-correction for multiple comparisons. Only electrodes that showed significantly larger mean amplitudes than the average were chosen. The amplitudes of the prime- and target-associated visual P1 and N1 were quantified at electrodes P7 and P8 (prime P1: 55–70 ms after target onset; prime N1: 95–110 ms after target onset; target P1: 155–170 ms after target onset) and at electrodes P9 and P10 (target N1: 240–255 ms after target onset). The N2 was quantified at electrode FCz (300–320 ms after target onset for incongruent flankers and 290–310 ms after target onset for congruent flankers) while the parietal P3 was quantified at electrodes PO1 and PO2 (285–305 ms after target onset). All averaged ERP components were quantified relative to the pre-stimulus baseline. The amplitudes of all these ERP peaks were quantified as mean amplitude values, which were averaged over their respective time windows. All ERP components were quantified on the single-subject level. For statistical analyses, amplitudes were averaged over all quantified electrodes whenever the quantification of a given peak took place at more than one electrode.

### Data-Driven Feature Extraction Procedure and Support Vector Machine (SVM) Analysis

Based on a median split of the prime compatibility effect (PCE) as our main behavioral performance parameter (i.e., RT difference between trials with compatible vs. incompatible primes), two groups of subjects were created: a “large PCE” group and a “small PCE” group. Importantly, this division of the sample helps us to determine which factors contribute to the size of the prime-induced conflict. Based on this, a machine learning approach was employed to classify/predict group membership on the basis of the neurophysiological data from correct trials, i.e., trials with correct responses between 100 and 1,000 ms. While it is well-known that a median split lowers experimental power and increases the risk of type I errors (Wicherts and Scholten, 2013), it is important to consider that a binary classification is a mandatory requirement for our machine learning approach (Kleinbaum et al., 2014). For classification, machine learning algorithms require a strict and objective criterion (Kleinbaum et al., 2014) like classifying individuals as either low or high performers. Given that psychological research on inter-individual performance differences can usually not provide fixed or objective cutoffs for classifying human behavior in a strictly categorical fashion, behavioral performance can only be judged as “good” or “bad” in relation to the performance of others. Thus, performance ratings in this field have to depend on what comparable individuals are capable of, resulting in an enforced categorization, e.g., by means of a median split.

Given a set of training data, each marked as belonging to the small or large PCE group, the SVM training algorithm builds a model that predicts whether a distinct feature falls into one category or the other. For an adequate prediction, therefore, a sufficient set of training data is pivotal. While there are no clear-cut recommendations comparable to a regular power estimation or the like, we would strongly advise to try to collect at least 200 data sets (i.e., have at least 100 subjects per group) when trying to predict a non-clinical/behavioral group membership on the basis of EEG data.

All time points from time point zero (target onset) to 1.5 s were extracted as possible features with the resolution of 256 Hz for each of the 60 channels and for every subject to determine the time-domain features (i.e., ERPs). Next, all features were normalized into a z-score. This was done to increase the convergence speed of feature detection algorithms (Theodoridis and Koutroumbas, 2011) and because features may bias the feature detection algorithm in case they have different value ranges because z-transformation makes all features have a mean of zero and a standard deviation equal to one, z-transformation circumvents this problem (Raschka, 2015). Next, we applied feature selection methods to eliminate surplus/irrelevant features. This is done to reduce the problem of having a ‘small’ data set relative to the size of the possible feature set, which could reduce classifier performance. In the feature selection stage, we selected an optimal subset of features from the original feature set. The feature selection algorithms can be roughly divided into two categories: “filter” and “wrapper” methods (Guyon and Eliseff, 2003). The filter methods select a subset of features according to general characteristics of the data, independently of the chosen classifier. To discriminate between classes, wrapper methods require a predetermined classifier and evaluate features according to their performance (Guyon and Eliseff, 2003). Because the selected features are based on classifier performance, wrapper methods usually lead to better results (Saeys et al., 2007), but are computationally slower than filter methods. To overcome this problem, we combined filter and wrapper methods using MATLAB 2017a (MathWorks Inc.): In a first step, filter methods are applied to select some features, which are then used as input for wrapper methods in a subsequent second step. In particular, *t*-test and sequential floating forward selection (SFFS) methods were employed as a filter and wrapper method, respectively (Saeys et al., 2007): First, a *t*-test was calculated to assess differences between the two PCE groups using the median split procedure for each time point (i.e., feature). A time point (feature) was selected (the precise *p*-values are given in Table 1 in the “Results” section) when the *p*-value was below 0.01. These selected features were then used as input for the SFFS algorithm. SFFS combines two separate algorithms (Chandrashekar and Sahin, 2014; Khazaee et al., 2016); i.e., sequential forward selection (SFS) and sequential backward selection (SBS). SFS starts from an empty set of features and sequentially adds features that result in the highest classifier accuracy when being combined with the features that have already been selected. SBS works in the opposite direction. In SFFS, each feature selection step includes both SFS and SBS (Chandrashekar and Sahin, 2014; Khazaee et al., 2016), which were implemented in MATLAB 2017a (MathWorks Inc.). Following SFFS, the selected features were fed to a SVM employing a radial basis function (RBF) kernel using MATLAB 2017a (MathWorks Inc.) and the LIBSVM toolbox. Importantly, the result of the SVM method was cross-validated in this study using the *k*-fold cross-validation procedure (Arlot and Celisse, 2010; Lee and Verri, 2002). Using the *k*-fold method data were randomly divided into *k* portions in which the *k* - 1 portion is considered as training data and the residual data is considered as testing data. By continuing this *k*-times, all subjects in the data set are alternately part of the testing and training set. The resulting classification accuracy is the average of all *k*-folds (Arlot and Celisse, 2010). Usually, the value of *k* is between 5 and 10 in machine learning. In this study, we used *k* = 10. Hence, there were 10 estimations of the predictability of behavioral performance for each extracted feature. Using these ten different estimations, the 99% confidence bounds were calculated for each feature using the data from the *k* = 10 estimations. These confidence bounds were then used to examine in how far the different features provided a significant increase in the predictability of behavioral performance. When there is no overlap between the calculated 99% confidence bounds we have a significant difference. While there is still a small risk for false positive features to survive feature selection and enter the machine learning approach, the subsequent *k*-fold validation procedure minimizes the risk of any false positive being selected as a predictive feature because it mixes and recombines the sample many times.

**Table 1:** Summary of the extracted features showing feature number, electrode site, time point in ms of the extracted feature after prime and target presentation, the mean predictability in percent and the significance as provided from the *t*-tests used as a filter method in the feature selection step.

(A) Compatible ERP features					

Feature	Electrode	Time point (ms after	Time point (ms after	Mean predictability (%)	Significance
number		prime onset)	target onset)		
1	TP10	63.9	3.9	62.78	0.00287
2	P12	450.6	390.6	66.53	0.00382
3	P4	306.1	246.1	69.44	0.04199
4	P10	251.4	191.4	69.89	0.02550
5	CP2	1517.0	1457.0	69.52	0.02182
6	F1	520.9	460.9	71.16	0.04783
7	F2	634.2	574.2	72.06	0.04025
8	CP4	1302.2	1242.2	73.69	0.02863
9	Fz	1462.3	1402.3	74.83	0.02027
10	P12	392.0	332.0	75.24	0.02130
11	P12	399.8	339.8	76.13	0.01541
12	P12	478.0	418.0	76.06	0.00919
13	CP3	1552.2	1492.2	76.09	0.02404
14	P10	243.6	183.6	76.09	0.01357
15	PO2	1356.9	1296.9	76.09	0.01885
16	CP4	1298.3	1238.3	77.34	0.03583
17	P12	497.5	437.5	77.78	0.00501
18	P12	376.4	316.4	77.36	0.02401
19	P7	75.6	15.6	76.98	0.04157
20	FT9	806.1	746.1	78.23	0.01155

**(B) Incompatible ERP features**					

1	PO2	67.8	7.8	64.56	0.00287
2	Fp1	1505.3	1445.3	67.36	0.00382
3	CP5	829.5	769.5	70.68	0.04199
4	PO1	1517.0	1457.0	74.36	0.02550
5	PO1	44.4	-15.6	73.98	0.02182
6	P9	1505.3	1445.3	74.01	0.04783
7	PO1	36.6	-23.4	74.02	0.04025
8	P9	1509.2	1449.2	74.02	0.02863
9	P10	56.1	-3.9	74.42	0.02027
10	O2	192.8	132.8	74.36	0.02130
11	P9	419.4	359.4	74.79	0.01541
12	O2	1106.9	1046.9	74.36	0.00919
13	AF4	1040.5	980.5	76.84	0.02404
14	CP5	1380.3	1320.3	76.86	0.01357
15	TP10	454.5	394.5	78.11	0.01885
16	CP5	1376.4	1316.4	78.09	0.03583
17	PO2	63.9	3.9	78.93	0.00501
18	O10	259.2	199.2	78.09	0.02401
19	P10	333.4	273.4	78.96	0.04157
20	PO1	52.2	-7.8	78.58	0.01155


### Source Localization Analysis

For each of the time-domain (ERP) features that were shown to be predictive for behavioral performance in the SVM analysis (see “Results” section) a source localization analysis was conducted. For this analysis, sLORETA (standardized low resolution brain electromagnetic tomography; Pascual-Marqui, 2002) was used. This procedure provides a unique solution to the inverse problem (Pascual-Marqui, 2002; Marco-Pallarés et al., 2005). For cortical origin sources sLoreta reveals high convergence with fMRI data and neuronavigated EEG/TMS studies, which underlines the validity of the estimated sources (Hoffmann et al., 2014; Dippel and Beste, 2015). For sLORETA, the intracerebral volume is partitioned into 6,239 voxels at 5 mm spatial resolution. The standardized current density at each voxel is calculated in a realistic head model (Fuchs et al., 2002) using the MNI152 template (Mazziotta et al., 2001). The voxel-based sLORETA images were compared between groups using the sLORETA-built-in voxel-wise randomization tests with 2,000 permutations, based on statistical non-parametric mapping (SnPM). Significant voxels (*p* < 0.01, corrected for multiple comparisons) were located in the MNI-brain.

### Statistics

For the behavioral and neurophysiologic analyses, only correct trials with RTs between 100 and 1,000 ms were included in order to exclude trials with premature responses and to reduce the effect of outliers on mean hit RTs. Separate repeated measures ANOVAs were performed to analyze behavioral and neurophysiological data. All ANOVAs used prime compatibility (compatible vs. incompatible) and flanker congruency (congruent vs. incongruent) as within-subject factors as well as the between-subject factor “PCE group” (large vs. small PCE). While the SVM does imperatively depend on this group dichotomization, we do recognize that the classical ERPs could also be analyzed by means of correlation/regression analyses. For the sake of completeness, we therefore provide those analyses as an add-on in the supplement for all interactions with the group factor. The degrees of freedom were adjusted using Greenhouse–Geisser correction and results were Bonferroni-corrected, whenever necessary. For all descriptive statistics, the mean value and standard error of the mean (SEM) are given as a measure of variability.

## Results

### Behavioral Data

#### PCE Groups

Before we split the data into two different PCE groups, we confirmed significant PCE effects (i.e., significantly faster responses in trials with compatible primes than in trials with incompatible primes) for the entire sample [*t*(242) = 24.412, *p* > 0.001; incompatible = 448 ms ± 0.30; compatible = 410 ms ± 2]. On average, the PCE (i.e., the hit RT difference between compatible and incompatible trials) was 38 ms ± 2. Independent-samples *t*-tests confirmed that the PCE groups (which were split at the median value of 35 ms) significantly differed in PCE magnitude [*t*(242) = 19.04, *p* < 0.001]: While the large PCE group had a condition difference of 57 ms (±2), the small PCE group had a mean condition difference of 19 ms (±1). There was no significant sex difference between groups (χ^2^ = 0.550; *p* = 0.458), with 82 females and 39 males in the large PCE group and 88 females and 34 males in the small PCE group. Also, there was no significant age difference between groups (χ^2^ = 3.703; *p* = 0.997). Given that the main focus of this study was the size of subliminally induced conflicts (which we operationalized via the PCE groups, we chose to only report the most relevant data (i.e., behavioral and neurophysiological measures showing relevant main effects and interactions involving the group factor) in the main manuscript. All other findings and results can be found in the Supplementary Material.

#### Speed–Accuracy Ratio

**FIGURE 2:**
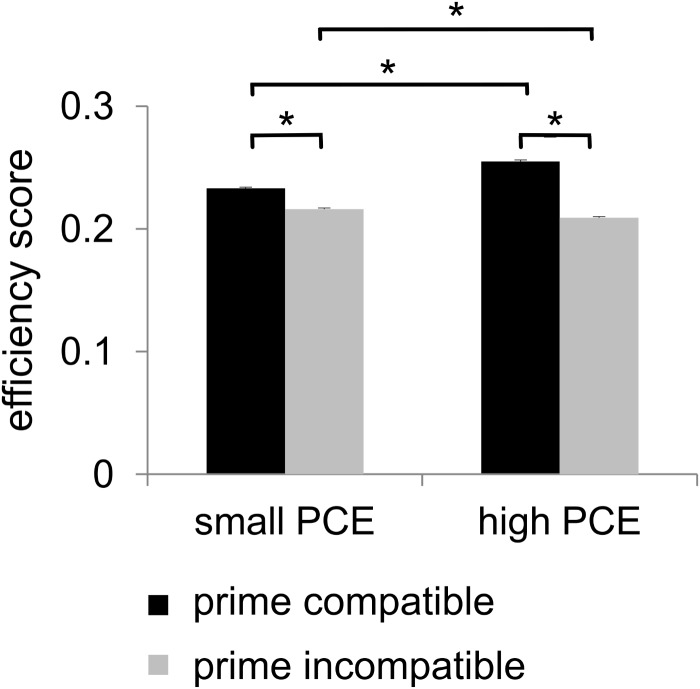
Behavioral data. There was a positive compatibility effect (PCE) for the efficiency score. It showed a main effect of prime compatibility (higher efficiency scores in case of compatible primes). Additionally, the efficiency score also showed an interaction of prime compatibility and PCE group as there was a larger priming effect/difference in the large PCE group compared to the small PCE group. Significant results (*p* ≤ 0.05) are denoted with an asterisk.

For the analysis of behavioral performance, we formed an efficiency score by dividing accuracy by mean hit RTs. Statistical analyses of this efficiency score revealed a main effect of prime compatibility [*F*(1,241) = 802.03, *p* < 0.001, $\eta_{p}^{2}$ = 0.769] with better performance in compatible (0.244 ± 0.001) than in incompatible (0.213 ± 0.001) trials. A main effect of flanker congruency [*F*(1,241) = 572.37, *p* < 0.001, $\eta_{p}^{2}$ = 0.704] indicated better performance in congruent trials (0.236 ± 0.001) than in incongruent trials (0.222 ± 0.001). An interaction of prime compatibility × PCE group was also found [*F*(1,241) = 183.74, *p* < 0.001, $\eta_{p}^{2}$ = 0.433] (see Figure 2). *Post hoc t*-tests revealed that there were compatibility effects in both the small PCE group [*t*(121) = 16.28; *p* < 0.001; incompatible = 0.216 ± 0.001 vs. compatible = 0.233 ± 0.001] and the large PCE group [*t*(120) = 23.41; *p* < 0.001; incompatible = 0.209 ± 0.001 vs. compatible = 0.255 ± 0.001]. Also, groups significantly differed in both compatible trials [*t*(241) = -8.08; *p* < 0.001] and incompatible trials [*t*(241) = 3.80; *p* < 0.001]. However, the PCE effect (compatible minus incompatible) was more pronounced in the large PCE group (0.045 ± 0.001) than in the small PCE group (0.016 ± 0.001) [*t*(177.98) = -13.52; *p* < 0.001]. Another significant interaction was found for flanker congruency × PCE group [*F*(1,241) = 9.55, *p* = 0.002, $\eta_{p}^{2}$ = 0.038] (see Figure 2). *Post hoc t*-tests demonstrated that there were congruency effects in both the small PCE group [*t*(121) = 17.353; *p* < 0.001; incongruent = 0.217 ± 0.001 vs. congruent = 0.232 ± 0.001] and the large PCE group [*t*(120) = 16.63; *p* < 0.001; incongruent = 0.226 ± 0.001 vs. congruent = 0.238 ± 0.001]. When separately compared, groups differed in performance in both congruent trials [*t*(241) = 2.53; *p* = 0.012] and incongruent trials [*t*(241) = 4.70; *p* < 0.001]. Furthermore, the flanker effect (congruent minus incongruent) was more pronounced in the small PCE group (0.015 ± 0.001) than in the large PCE group (0.011 ± 0.001) [*t*(230.83) = -3.095; *p* < 0.002]. Further investigating the interaction of flanker congruency × PCE group, we found that behavioral PCE size and flanker effect were negatively correlated [*r* = -0.192, *p* = 0.003]. All other main effects and interactions of the speed–accuracy ratio analyses involving the PCE group were not significant (all *F* ≤ 2.99; *p* ≥ 0.085).

#### Summary of Behavioral Data

In summary, the behavioral data showed a PCE in the efficiency score, which could also be observed in both accuracy and hit RTs, when separately analyzed (see Supplementary Material). Most importantly, there was an interaction of prime compatibility and PCE group in all three types of behavioral measures. This interaction was driven by the fact that the overall performance (and not just RTs) showed a larger priming effect/difference in the large PCE group as compared to the small PCE group.

### Neurophysiological Data

#### Prime N1

**FIGURE 3:**
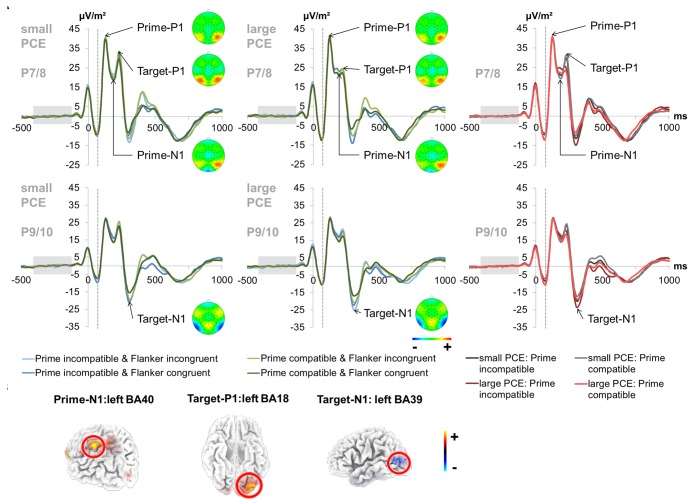
Early attentional ERPs. **(A)** Prime-locked P1 and N1 ERPs elicited by the prime stimulus (onset at time point zero) and target stimulus (onset at 60 ms) at electrodes P7 and P8 (pooled) for the prime P1, prime N1 and target P1 and at electrodes P9 and P10 (pooled) for the target N1. The dashed vertical line in the plot shows the target onset. In the left and middle column, each combination of prime compatibility and flanker congruency is depicted separately for each group (small vs. large PCE effect) (blue denotes incompatible primes and green denotes compatible primes while lighter shades of the respective color denote incongruent flankers and darker shades denote congruent flankers). Topography maps of the peaks are depicted right next to the respective peak names. In the right column, data from the large and small PCE groups are displayed together with waveforms pooled across flanker conditions (gray denotes the small PCE group and red the large PCE group while lighter shades of the respective color denote compatible primes and darker shades denote incompatible primes). Please note that amplitudes are given in μV/m^2^ due to the CSD interpolation (cf. “Materials and Methods” section). **(B)** The sLORETA plots (corrected for multiple comparisons using SnPM) show the source of group difference in the magnitude of condition effects which was based on activation differences within the left BA40 (inferior parietal lobule/TPJ) at the time point of the prime-N1, left BA18 (inferior occipital gyrus/V2) at the time point of the target-P1, and left BA39 (middle temporal gyrus/angular gyrus/TPJ) at the time point of the target-N1.

The prime- and target-associated P1 and N1 ERPs are illustrated in Figure 3.

The repeated-measures ANOVA for the prime-associated N1 (95–110 ms after target onset; pooled across electrodes P7 and P8) revealed a significant main effect of prime compatibility [*F*(1,241) = 59.13, *p* < 0.001, $\eta_{p}^{2}$ = 0.197] with smaller amplitudes in incompatible (23.99 μV/m^2^ ± 1.36) than in compatible (22.31 μV/m^2^ ± 1.34) trials. A main effect of flanker congruency [*F*(1,241) = 4.02, *p* = 0.046, $\eta_{p}^{2}$ = 0.016] indicated smaller amplitudes in incongruent (23.35 μV/m^2^ ± 1.33) than in congruent (22.95 μV/m^2^ ± 1.37) trials. Also, an interaction of prime compatibility × PCE group [*F*(1,241) = 5.15, *p* = 0.024, $\eta_{p}^{2}$ = 0.021] was found. *Post hoc t*-tests revealed significant compatibility effects in both the small PCE group [*t*(121) = -4.30; *p* < 0.001; incompatible = 23.38 μV/m^2^ ± 1.93 vs. compatible = 22.20 μV/m^2^ ± 1.92] and the large PCE group [*t*(120) = -6.40; *p* < 0.001; incompatible = 24.60 μV/m^2^ ± 1.92 vs. compatible = 22.43 μV/m^2^ ± 1.87]. Additionally, groups did not differ in amplitude in compatible or incompatible trials (both *p* > 0.656). However, amplitude differences (compatible minus incompatible) were more pronounced in the large PCE group (2.17 μV/m^2^ ± 0.33) than in the small PCE group (1.18 μV/m^2^ ± 0.27) [*t*(230.69) = 2.26; *p* = 0.024]. Source localization via sLORETA revealed that this group difference in the magnitude of condition effects was associated with activation differences in the left BA 40 (inferior parietal lobule/TPJ). In this context, please note that the prime-associated N1 values were above zero due to the temporal location of the baseline interval. As a consequence, larger absolute values may still constitute smaller amplitude. Further investigating the interaction of prime compatibility × PCE group, we found a negative correlation between behavioral PCE size and the priming effect (i.e., prime condition difference) on the prime-N1 [*r* = 0.369, *p* < 0.001]. All other main effects and interactions were not significant for prime N1 amplitudes (all *F* ≤ 1.19; *p* ≥ 0.275).

#### Target P1 and Target N1

For the target-associated P1 (155–170 ms after target onset; pooled across electrodes P7 and P8), there was a significant main effect of prime compatibility [*F*(1,241) = 14.56, *p* < 0.001, $\eta_{p}^{2}$ = 0.057] with larger amplitudes in incompatible (27.79 μV/m^2^ ± 1.53) than in compatible (26.90 μV/m^2^ ± 1.50) trials. There was also a significant main effect of flanker congruency [*F*(1,241) = 126.66, *p* < 0.001, $\eta_{p}^{2}$ = 0.345] with larger amplitudes in incongruent (28.63 μV/m^2^ ± 1.53) than in congruent (26.07 μV/m^2^ ± 1.50) trials. There was an interaction of prime compatibility × PCE group [*F*(1,241) = 11.31, *p* = 0.001, $\eta_{p}^{2}$ = 0.045]. Source localization via sLORETA revealed that group differences in the magnitude of condition effects were associated with activation differences in the left BA 18 (inferior occipital gyrus/V2). Furthermore, there was an interaction of prime compatibility × flanker congruency × PCE group [*F*(1,241) = 6.58, *p* = 0.011, $\eta_{p}^{2}$ = 0.027]. We further investigated the latter by conducting separate analyzes for the small and large PCE group. In the small PCE group, there was an interaction of prime compatibility × flanker congruency [*F*(1,121) = 13.83, *p* < 0.001, $\eta_{p}^{2}$ = 0.103]. Further analyses revealed significant differences for all possible contrasts in the small PCE group (all *p* < 0.001). Yet, the PCE (i.e., incompatible–compatible) was smaller in trials with incongruent flankers (-0.97 μV/m^2^ ± 0.39) than in trials with congruent flankers (1.18 μV/m^2^ ± 0.45) [*t*(242) = 3.71; *p* < 0.001]. Likewise, the flanker congruency effect (i.e., incongruent–congruent) was smaller in incompatible primes (1.68 μV/m^2^ ± 0.42) than in compatible primes (3.84 μV/m^2^ ± 0.42) [*t*(242) = 3.71; *p* < 0.001]. In the large PCE group, there was no such interaction of prime compatibility × flanker congruency [*F*(1,120) = 0.075, *p* = 0.785, $\eta_{p}^{2}$ = 0.001]. All other main effects and interactions were not significant for target P1 amplitudes (all *F* ≤ 3.53; *p* ≥ 0.061).

The analysis of the target-associated N1 (240–255 ms after target onset; pooled across electrodes P9 and P10) revealed a significant main effect of prime compatibility [*F*(1,241) = 87.38, *p* < 0.001, $\eta_{p}^{2}$ = 0.266] with larger amplitudes in incompatible (-20.88 μV/m^2^ ± 1.38) than in compatible (-17.87 μV/m^2^ ± 1.36) trials. A main effect of flanker congruency [*F*(1,241) = 91.52, *p* < 0.001, $\eta_{p}^{2}$ = 0.275] indicated larger amplitudes in incongruent (-20.84 μV/m^2^ ± 1.38) than in congruent (-17.91 μV/m^2^ ± 1.36) trials. Moreover, an interaction of prime compatibility × PCE group [*F*(1,241) = 31.82, *p* = 0.001, $\eta_{p}^{2}$ = 0.117] was found. *Post hoc t*-tests revealed that there were compatibility effects in both the small PCE group [*t*(121) = -3.45; *p* = 0.001; incompatible = -18.29 μV/m^2^ ± 1.83 vs. compatible = -17.10 μV/m^2^ ± 1.81] and the large PCE group [*t*(120) = -8.86; *p* < 0.001; incompatible = -23.47 μV/m^2^ ± 2.07 vs. compatible = -18.64 μV/m^2^ ± 2.04]. When directly compared, groups did not differ in amplitude in compatible or incompatible trials (both *p* > 0.063). Yet, the PCE (compatible minus incompatible) was more pronounced in the large PCE group (4.82 μV/m^2^ ± 0.54) than in the small PCE group (1.19 μV/m^2^ ± 0.34) [*t*(203.57) = -5.63; *p* = 0.001]. Source localization via sLORETA revealed that this group difference in the magnitude of condition effects was associated with activation differences in the left BA 39 (middle temporal gyrus/angular gyrus/TPJ). Further investigating the interaction of prime compatibility × PCE group, we found a positive correlation between behavioral PCE size and the target-N1 priming effect [*r* = 0.398, *p* < 0.001]. All other main effects and interactions involving the PCE group were not significant for target N1 amplitudes (all *F* ≤ 0.417; *p* ≥ 0.519).

#### N2

**FIGURE 4:**
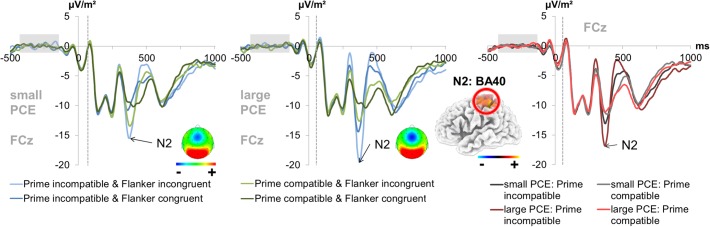
Fronto-central N2. The graphs separately depict the N2 peak for each group (small vs. large PCE effect) at electrode FCz. The dashed vertical line in the plot shows the target onset. The topography maps of the PCE effect differences (i.e., the difference between compatible and incompatible trials at the N2 peak of each group) are depicted in the respective graphs. In the left and middle column, each combination of prime compatibility and flanker congruency (blue denotes incompatible primes and green denotes compatible primes while lighter shades of the respective color denote incongruent flankers and darker shades denote congruent flankers) is depicted for both groups. In the right column, data from large and small PCE groups are displayed together with waveforms pooled across flanker conditions (gray denotes the small PCE group and red the large PCE group while lighter shades of the respective color denote compatible primes and darker shades denote incompatible primes). Please note that amplitudes are given in μV/m^2^ due to the CSD interpolation (refer “Materials and Methods” section). The sLORETA plots (corrected for multiple comparisons using SnPM) show the source of group difference in the magnitude of condition effects which was based on activation differences within the left BA40 (inferior parietal lobule/TPJ).

For the fronto-central N2 amplitude (300–320 ms after target onset for incongruent flankers and 290–310 ms after target onset for congruent flankers) at electrode FCz (see Figure 4), there was a main effect of prime compatibility [*F*(1,241) = 155.87; *p* < 0.001; $\eta_{p}^{2}$ = 0.393] with larger amplitudes in incompatible (-14.86 μV/m^2^ ± 0.91) than in compatible (-11.17 μV/m^2^ ± 0.88) trials. There was also a significant main effect of flanker congruency [*F*(1,241) = 173.88; *p* < 0.001; $\eta_{p}^{2}$ = 0.419] with larger amplitudes in incongruent (-15.06 μV/m^2^ ± 0.92) than in congruent (-10.97 μV/m^2^ ± 0.87) trials. Also, an interaction of prime compatibility × PCE group [*F*(1,241) = 43.71; *p* < 0.001; $\eta_{p}^{2}$ = 0.154] was found. *Post hoc t*-tests showed that there were compatibility effects in both the small PCE group [*t*(121) = -4.90; *p* < 0.001; incompatible = -13.00 μV/m^2^ ± 1.12 vs. compatible = -11.27 μV/m^2^ ± 1.09] and the large PCE group [*t*(120) = -11.90; *p* < 0.001; incompatible = -16.73 μV/m^2^ ± 1.43 vs. compatible = -11.08 μV/m^2^ ± 1.38]. When separately compared, groups did not differ in amplitude in compatible or incompatible trials (both *p* > 0.430). Yet, the PCE (compatible minus incompatible) was more pronounced in the larger PCE group (5.64 μV/m^2^ ± 0.47) than in the small PCE group (1.73 μV/m^2^ ± 0.35) [*t*(222.621) = -6.60; *p* < 0.001]. Further investigating the interaction of prime compatibility × PCE group, we found a positive correlation between behavioral PCE size and the N2 priming effect [*r* = 0.369, *p* < 0.001]. Source localization via sLORETA revealed that this group difference in the magnitude of condition effects was associated with activation differences in the left BA 40 (inferior parietal lobule/TPJ).

All other main effects and interactions involving the PCE group were not significant for N2 amplitudes (all *F* ≤ 1.98; *p* ≥ 0.160).

#### P3

For the parietal P3 amplitude (285–305 ms after target onset; pooled across electrodes PO1 and PO2), none of the investigated factors or interactions reached significance (all *F* ≤ 1.04, *p* ≥ 0.307) (see Supplementary Figure S2).

#### Summary of Neurophysiological Data

With respect to the interaction of PCE group and priming effects observed at the behavioral level, we found corresponding effects for several ERP amplitudes. Amplitude differences (compatible minus incompatible) were more pronounced in the large PCE group than in the small PCE group for the prime-N1, target-N1 and N2.

### Machine Learning Analysis

**FIGURE 5:**
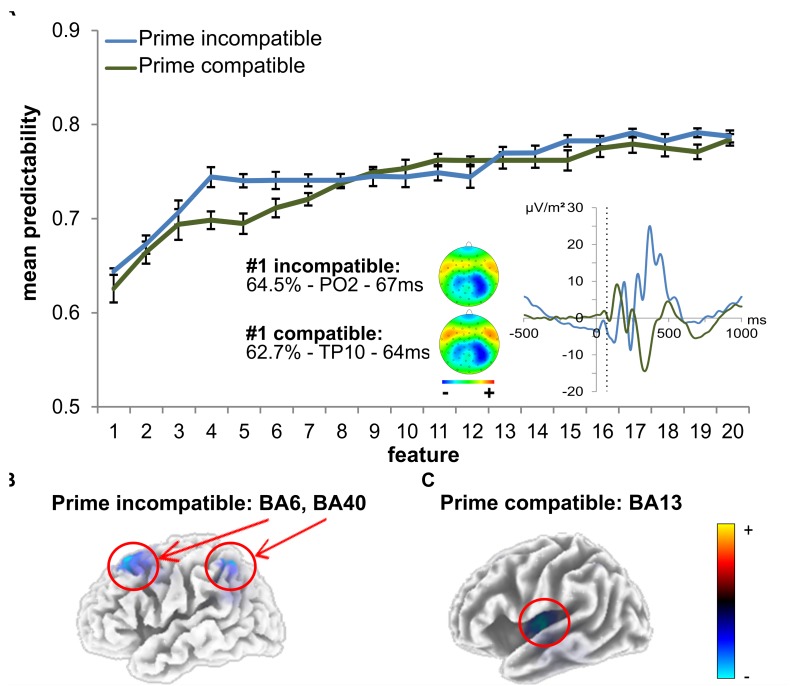
SVM data using time domain ERP data. **(A)** The mean predictability is given depending on the number of features. The curves show the mean predictability (blue for incompatible, green for compatible) and the 99% confidence level bounds (error bars). For the first feature, the prime-locked ERP curve is also shown (blue for incompatible, green for compatible). The dashed vertical line in the plot shows time points of features at electrodes PO2 (incompatible) and TP10 (compatible). The scalp topography plots reveal the distribution of voltages for this feature. **(B)** The sLORETA plots (corrected for multiple comparisons using SnPM) show the source of the signal at the time point of the feature in the incompatible prime condition, which was based on activation differences within BA6 (middle frontal gyrus) and BA40 (left inferior parietal gyrus/TPJ) (incompatible). **(C)** The sLORETA plots (corrected for multiple comparisons using SnPM) show the source of the signal at the time point of the feature in the compatible prime condition, which was based on activation differences within BA13 (left insula) (compatible).

As outlined in the “Materials and Methods” section, the *k*-fold method (*k* = 10) was used to evaluate the predictability of behavioral performance using ERP data. For each extracted feature, there were 10 varying estimations of the predictability of behavioral performance. Using the data from these *k* = 10 estimations, 99% confidence bounds were calculated for each feature. A significant difference is indicated by no overlap between the calculated 99% confidence bounds of two features. The results of the analysis using ERP data are shown in Figure 5. Error bars represent the 99% confidence bounds. Importantly, group difference were separately assessed for the ERPs in the prime compatible and incompatible conditions due to the constraints of the applied method. Table 1 shows a detailed summary of the ERP-features selected by the feature extraction approach.

In prime compatible trials, there was one ERP feature which significantly increased predictability of behavioral performance (i.e., PCE group membership). This ERP feature (3 ms after target onset at electrode TP10) led to a classification/prediction accuracy of ∼63%, which is significantly different from chance level as indicated by the 99% confidence bounds. Adding more features still led to a numerical increase in classification/prediction accuracy (see Figure 5 and Table 1), but this increase was not statistically significant, as the 99% confidence bounds largely overlapped with the classification/prediction accuracy obtained after adding the second feature. An sLORETA analysis shows that PCE group differences in the compatible condition were located in the left insula (BA13) at the time point of the predictive feature.

For the prime incompatible trials, the first ERP feature (7.8 ms after target onset at electrode PO2) provided a predictability of ∼64% (see Figure 5 and Table 1). As can be seen in Figure 5, the addition of further features numerically increased predictability, but this was not significant given the overlap of the 99% confidence bounds for each of the following added features. The sLORETA analysis revealed that PCE group differences were located in the MFG (BA6), between the SMA and FEF and in the left inferior parietal gyrus/TPJ (BA 40), at the time point of the predictive feature. A second peak was observed in the left inferior parietal gyrus/TPJ (BA 40). To confirm the relevance of our selected features, we also determined the predictability of the regular ERP components which we identified to distinguish attentional and control-related processes (prime-P1and N1, target-P1 and N1, N2 and P3) at the pre-defined electrodes and time windows (see previous results on classical ERPs). For the prime compatible trials, ERP components had a predictability of ∼56%, and for incompatible trials ERP components led to a classification/prediction accuracy of∼61%. This means that both classical ERP components had a lower predictability than the feature selected by SVM.

## Discussion

Subliminally perceived information evokes a different kind of response conflict than consciously processed information (Boy et al., 2010b). Yet still, only very little is known about how subliminally triggered response conflicts emerge, which factors contribute to the size of this kind of conflict (i.e., how well someone is able to control/inhibit incorrect automatic response tendencies), and whether this inter-individual variability also relates to differences in the size of consciously perceived conflicts. We assessed conflict size as an individual feature using a modified response conflict paradigm (Stock et al., 2016a) and quantified attentional processes, conflict monitoring, and S–R mapping with the help of ERP analyses. As classical ERP analyses are often limited to a small number of neurophysiologic features and are only correlative in nature, they, however, do not allow to reasonably classify/predict behavioral performance, including the size of conflicts. In order to overcome those limitations and identify the cognitive sub-processes that best classify/predict the size of subliminally induced response conflicts, we additionally applied a purely data-driven machine learning approach on the neurophysiological data, which accounts for all electrodes and time points, irrespective of whether or not they represent a “classical” ERP. In combination with source localization techniques, this approach allows to derive new, testable hypotheses about which cognitive sub-processes and brain regions likely classify/predict (and thus potentially cause) differences in the size of subliminally triggered response conflicts.

### Behavioral Data and Standard ERP Data

In line with previous studies, we found that both consciously and subliminally induced conflicts produced significant behavioral performance impairments, which worsened in a non-additive way when the respective other kind of conflict was also present (Stock et al., 2016a) (for a discussion of this interaction please refer the Supplementary Material). Most importantly, however, we found that the size of subliminally induced response conflicts (as operationalized by the large and small PCE group) was not limited to the domain of response times, which had been used to form the two groups. Instead, the PCE seemed to be a general modulator of performance, as reflected by our behavioral efficiency score. This even extended to the consciously perceived conflicts, albeit in a rather unexpected direction: Individuals who showed a larger behavioral modulation by subliminal primes (i.e., a larger PCE) also showed a smaller behavioral modulation by the consciously perceived distractors (i.e., a smaller Flanker effect). This suggests that even though the PCE is a rather specific measure, it probably reflects a general tendency for processing task-irrelevant information differently. This interpretation is well in line with previous studies which stated that active task sets may tune sensorimotor pathways to enable a strongly automated processing of task relevant cues, even if those are only subliminally presented (Muhle-Karbe et al., 2017). Subliminal input may hence bias early information accumulation and activation in decision circuits (Leuthold and Kopp, 1998; Vorberg et al., 2003; Parkinson and Haggard, 2014), which leads to the automatic activation of response tendencies that may ultimately facilitate (correct) responses in case of compatible primes (Eimer and Schlaghecken, 1998, 2003; Eimer, 1999; Schlaghecken and Eimer, 2000) (Further discussion of the reproduction of the general interaction between primes and flankers on the behavioral level can be found in the Supplementary Material). *Given that we also found the size of subliminally triggered response conflicts to be related to that of consciously processed ones, it seems possible that our two performance groups might reflect general inherent differences in the strength of task sets, which determine how much task-irrelevant information is attended/processed and subsequently converted into automatic response tendencies. Yet, it seems that the tendency to attend and process subliminal information more thoroughly may increase subliminal conflicts (as operationalized by PCE groups), and at the same time diminish conflict size when the distractor is consciously perceived. While this finding is indeed very interesting, we could not find any theoretical framework that suggests a comprehensible explanation for this observation.*

In order to separate and identify the cognitive-neurophysiological sub-processes that might explain the size of subliminal response conflicts, we analyzed neurophysiological data using both “traditional” ERP analyses and a data-driven machine learning approach. In this context, we decided to primarily focus on the interaction of prime compatibility and PCE group as the size of priming effects across groups clearly distinguishes the two performance groups and best reflects the magnitude of subliminally induced response conflicts. As subliminal priming may bias early information accumulation (Leuthold and Kopp, 1998; Vorberg et al., 2003; Parkinson and Haggard, 2014), we assessed early attentional stimulus processing by quantifying the prime- and target-associated P1 and N1 components (Luck et al., 2000). We found that the size of behavioral subliminal response conflicts was reflected by the size of condition differences in the initial processing of both subliminally and consciously perceived stimuli. Additionally, early attentional processing of subliminal information, as reflected by the prime-associated N1 amplitude, was enhanced in compatible trials. This nicely matches the hypothesis that individuals, who show a more pronounced priming effect, benefit from enhanced attentional processing of the compatible subliminal primes (Luck et al., 2000; Herrmann and Knight, 2001; Mahé et al., 2014; Nicholls et al., 2015). It is furthermore in line with the notion that the intensity of early attention allocation and attentional processing of the target and comparable distractor stimuli determines the size of subliminal response conflicts (Naccache et al., 2002). On the neuroanatomical level, we found this effect to be reflected in the activity of the inferior parietal cortex/TPJ. This finding nicely matches the available literature as this brain area is an integral part of frontoparietal networks that contribute to both attentional stimulus processing and task set representation (Kiefer, 2008; Kiefer et al., 2011; Vossel et al., 2014; Muhle-Karbe et al., 2017): With respect to task sets, this brain region has been shown to be involved in the processing of task rules/S–R mappings and to contribute to the selective tuning of sensory regions according to task demands (Crone et al., 2006; Loose et al., 2017; Muhle-Karbe et al., 2017). Regarding attentional processing, the TPJ has been shown to reflect attentional re-orienting as well as feature-based attention and has been suspected to potentially hamper goal-directed behavior, when not properly suppressed (Liu et al., 2011; Vossel et al., 2014).

In contrast to the prime-associated N1, we found the amplitudes of target-associated P1 and N1 components to be increased in case of subliminal conflicts. These two ERP components have been suggested to reflect early attentional processing of stimulus features (Luck et al., 2000) and may therefore also reflect priming (Buckner et al., 1998), as attentional processing of the prime seems to be the determining factor thereof. Importantly, our findings of (relatively) smaller target-associated P1 and N1 amplitudes match studies which report that primed or repeated features elicit smaller, more adapted neuronal responses (Schacter et al., 2007). Additionally, it could also be argued that enhanced processing of consciously perceived information after incompatible subliminal input reflects a first, compensatory strategy in order to deal with incompatible automatic response tendencies. Still, further studies will be needed to substantiate this claim. On the neuroanatomical level, we found the effects in the target-P1, which also reflected group dependent differences in flanker effect size, and the N1 to be reflected by the left V2 and the left angular gyrus, respectively. V2 is a secondary visual cortex/visual association area and one of the “starting points” of attention networks (Vossel et al., 2014). It is typically concerned with processing different visual properties of stimuli, including the orientation of stimuli (which was the task-relevant stimulus property in our paradigm) (Anzai et al., 2007) and may help to filter out relevant stimuli among various visual input (Shipp et al., 2009). V2 may furthermore have been modulated/biased by top-down activity from frontoparietal areas, likely including task sets (Vossel et al., 2014). The angular gyrus is adjacent to the inferior parietal cortex and known to play a key role for spatial cognition and attention, left-right orientation, and orientation toward salient or task-relevant features (Seghier, 2013).

Lastly, we also found the behavioral differences in subliminal response conflicts to be reflected by fronto-central N2 amplitudes, which are known to reflect cognitive conflict and the effort associated with a given task (Botvinick et al., 2004; Folstein and Van Petten, 2007). We did not only find the typical enhancement N2 amplitudes, which signals enhanced control and effort in case of response conflicts, but we also found the size of subliminal response conflicts to be reflected by the size of modulation in this process/ERP (i.e., we found more pronounced condition differences in the large PCE group than in the small PCE group). This underlines the notion that subliminal response conflicts may still modulate the degree of response conflict and cognitive effort (Botvinick et al., 2004; Folstein and Van Petten, 2007). We did not find the source of prime-related N2 differences in the ACC, which is most commonly identified as the source of differences in conscious, conflict monitoring (Botvinick et al., 2004; Larson et al., 2014). This, however, matches previous studies that have already shown that masked subliminal primes do not seem to modulate ACC activity, even when eliciting significant effects (Desender and Van den Bussche, 2012). Instead, our effect was again based on activation differences in the left inferior parietal cortex/TPJ, which reflects both task set activation and attentional processing of task-related features (Vossel et al., 2014; Muhle-Karbe et al., 2017). We, however, found no modulation as the level of response selection and stimulus–response mapping, as reflected by the P3 component (Verleger et al., 2015).

### Machine Learning Data

In the context of machine learning, the terms of classification and prediction are used to describe how well an algorithm is able to correctly allocate individual datasets to distinct categories, based on the variation found within those datasets. In the context of our study, this translates to the prediction or classification of our median-split groups (small vs. large PCEs) on the basis of the available neurophysiological data, where each data point is treated as a distinct feature. When we applied the data-driven machine learning approach to our neurophysiological data, we found that in each priming condition, there was only one feature that significantly predicted inter-individual behavioral performance differences in the size of the subliminally triggered response conflict (i.e., group membership) with 63 to 64% accuracy. Importantly, both predictive ERP features were found in the time range of very early attentional processing (∼63–67 ms after prime onset and ∼3–7 ms after target onset). Given that it is impossible to sufficiently process target information within 3 to 7 ms, it can be safely concluded that the size of subliminal response conflicts between prime and target is driven by how much attention was devoted to the prime. This prime processing then drove the resulting conflict between prime- and target-associated response tendencies, i.e., the mismatch and subsequent conflict between response tendencies that were driven by subliminal vs. conscious stimulus processing. This also implies that intra-individual variations in the size of subliminal conflicts cannot be (mainly) due to differences in how well a person can perform response selection or shield task goals (Goschke and Dreisbach, 2008; Beste et al., 2017). This means variations in timing (i.e., selectively attending the target, but not the prime that precedes it), or an increase in shielding upon the detection of a conflict cannot explain the observed priming effects. Instead, the size of subliminal response conflicts most likely arises from how strongly top-down task set representations bias/enhance the automatic processing of task-relevant stimulus features. In other words, it seem to be the allocation of cognitive resources like attention to initial stimulus processing, which determines conflict size (Naccache and Dehaene, 2001). Source localization of the predictive features showed that individuals with larger subliminal response conflicts showed increased activation in the left insula (LI), the MFG and, albeit to a lesser degree, the inferior parietal cortex/TPJ. The insula is connected to both subcortical and cortical regions and has been suggested to be “a key brain region for the integration of conscious and non-conscious processing” (Meneguzzo et al., 2014). The insula has furthermore been shown to reflect task set representation (Crone et al., 2006). The involvement of MFG matches this picture as it is involved in the interruption of ongoing endogenous attentional processes and helps to reorient attention to an exogenous stimulus (Japee et al., 2015). Hence, this region likely plays a key role in determining how much subliminal information is attended. This further matches the finding that MFG activity was strongest in between the SMA and FEF. The SMA may increase coupling between visuomotor brain regions, thus reflecting a higher efficiency of unconscious processing (Ulrich and Kiefer, 2016) and has repeatedly been demonstrated to reflect effects of subliminal priming (Boy et al., 2010a; D’Ostilio et al., 2012). The FEF, on the other hand, is an integral part of the dorsal attention system, which has been suggested to modulate activity in visual areas and which plays a role in feature-based attention (Vossel et al., 2014). Lastly, we again also found a minor activation in the left inferior parietal cortex/TPJ, which plays a pivotal role in feature-based attention reorienting, as well as task set representations (Crone et al., 2006; Vossel et al., 2014; Loose et al., 2017; Muhle-Karbe et al., 2017). Taken together, the identified activity differences in those brain regions further underpin the notion that the size of subliminal response conflicts might reflect a general tendency of how much subliminal information is attended, based on the strength of top-down task sets. This is well in line with the notion of prepared reflexes, according to which top-down processes increase responsiveness to task-relevant stimulus features in both primary sensory areas and sensory association cortices and thus enable an unconscious, rather automatic feature processing (Kiefer, 2008; Kiefer et al., 2011; Muhle-Karbe et al., 2017). This early information accumulation is followed by activation in decision circuits (Leuthold and Kopp, 1998; Vorberg et al., 2003; Parkinson and Haggard, 2014), which ultimately results in an automatic response tendency (Eimer and Schlaghecken, 1998, 2003; Eimer, 1999; Schlaghecken and Eimer, 2000).

At first glance, the findings of our SVM approach might be considered to be at odds with the “classical” ERP results as well as other studies that have repeatedly identified fronto-central ERP components as the most reliable correlate of cognitive control, conflict and effort (Larson et al., 2014). Yet, it must be considered that we set out to identify predictors/classifiers, not correlates of subliminal response conflicts. This means that even though the size of a subliminal response conflicts is reflected by the N2 component and other ERPs, differences in the degree of conflict or monitoring capacities are not what initially seems to give rise to the observed behavioral differences. Instead, it seems that all observed ERP differences are (at least partly) caused by differences in a preceding event, namely the degree of initial feature processing, which is independent of conflicts between response options and instead depends on the modulation by top-down task sets.

### Limitations

A potential limitation of our study is that we used identical positions for masked primes and visible targets, but different positions for visible flankers in combination with a successive presentation of masked prime and visible targets in contrast to the concomitant presentation of visible flankers and primes. While we used a well-established paradigm with stably reproducible effects, this might have potentially had an influence on attentional and perceptual processes.

Related to this, is should be mentioned that the close temporal proximity between prime and target has likely produced some overlapping of prime- and target-associated cognitive sub-processes, even though both stimuli produced temporally distinct attentional ERPs (i.e., P1 and N1) and we furthermore found differential effects on those components. The traditional labeling of quantified ERP components (especially the target-associated P1 and N1) may therefore be criticized as somewhat oversimplified. In this context, it is, however, also important to note that all conditions had the same temporal setup, so that they may be compared with each other even in case of temporal ERP component overlap (for a more detailed discussion of this issue, please see Zink et al., 2019).

Lastly, there are currently only few studies investigating the interaction of subliminally and consciously triggered conflicts. In this context, the scarcity of other available paradigms demonstrating the interaction of subliminally and consciously perceived distractors in general demands for further research.

### Outlook

Our study focused on the question of how the size of response conflicts triggered by subliminal primes comes about, but it may be interesting to also use SVM to investigate whether the mechanism underlying consciously processed flanker conflicts is similar, or even comparable. Given that we found the two PCE groups to also differ with respect to flanker effect size, it could be conceivable that at least some aspects of this conflict have the same origin, namely increased attention to task-relevant features in all stimuli, including distractors. The finding that individuals in the larger PCE group did, however, show smaller flanker effects, and not larger ones, does not quite match this simple explanation: If both kinds of conflicts (prime- and flanker-induced) were merely due to attention allocation to task-relevant stimulus properties, we should have seen larger flanker effects in the large PCE groups (and not vice versa). The finding of a negative correlation between prime and flanker conflict size suggests that the conflict induced by consciously perceived flankers cannot be solely driven by the same factors as the conflict induced by subliminal primes. This could potentially be due to the fact that the flankers are consciously processed and conflicts may therefore be detected, thus triggering additional top-down processing. Yet, the difference between prime and flanker conflict size could also be associated with stimulus location (see “Limitations” section). Investigating all of those speculations will, however, require further research.

## Conclusion

In summary, we investigated how subliminally triggered response conflicts emerge and which factors contribute to the size of this kind of response conflict. While conflict size is associated with conflict monitoring/effort as well as early attentional stimulus processing, a data driven machine learning procedure demonstrated that only the initial processing of subliminal information is actually predictive of conflict size. Specifically, we could show that the size of subliminally induced response conflicts is determined by how much subliminal task-irrelevant information is attended/processed, which most likely determines the intensity the automatic response tendencies it triggers. This was reflected by activity modulations in left frontoparietal and attentional networks including several the insula, MFG, angular gyrus and inferior parietal cortex/TPJ. Those brain regions play a key role in determining how much subliminal information is attended based on pre-active top-down task sets. Taken together, our analyses have shown that even though response conflicts reliably modulate fronto-central conflict monitoring processes, their size is not initially determined or caused by the mismatch between task-relevant and task-irrelevant response tendencies. Instead, the size of response conflicts seems to be rooted in the intensity with which an individual initially attends and processes task-relevant distracting information, which ultimately results in differences in automatic response tendency generation.

## Author Contributions

All authors were involved in designing the study and collecting the data. WB and A-KS collected the data. WB, AV, and A-KS analyzed the data. All authors were included in writing the manuscript and approved its final version.

## Conflict of Interest Statement

The authors declare that the research was conducted in the absence of any commercial or financial relationships that could be construed as a potential conflict of interest.

## Footnotes

1 Please note that throughout the entire manuscript, the term “prediction” will be strictly used in the context provided by machine learning approaches. This is, how well can individuals with “good” or “bad” behavioral performance be distinguished with the help of a given neurophysiological feature.
