# Supplementary material for: The Intensity of Early Attentional Processing, but Not Conflict Monitoring, Determines the Size of Subliminal Response Conflicts
Source: Front Hum Neurosci. 2019 Feb 20;13:53. doi: 10.3389/fnhum.2019.00053 (PMC6391363; doi:10.3389/fnhum.2019.00053)
Supplement: Supplementary file 1 [file Data_Sheet_1.pdf]

Supplementary Material

**Journal**	Frontiers in Human Neuroscience
**Title**	The intensity of early attentional processing, but not conflict monitoring, determines the size of subliminal response conflicts
**Authors**	Bensmann W, Vahid A, Beste C, Stock AK
**Correspondence**	Ann-Kathrin.Stock@uniklinikum-dresden.de

Behavioral data: Accuracy

The repeated-measures ANOVA for accuracy (percentage of correct responses) revealed a main effect of prime compatibility [F(1,241) = 229.36, p < .001, η_p^2 = .488] with a larger accuracy in compatible (99.10% ± .08) than in incompatible (95.24 % ± .28) trials. Additionally, a significant main effect of flanker congruency was found [F(1,241) = 131.12, p < .001, η_p^2 = .352]. Participants responded less accurately in incongruent trials (96.34 % ± .21) than in congruent trials (97.99 % ± .14). An interaction of prime compatibility x PCE group was also found [F(1,241) = 36.12, p < .001, η_p^2 = .130] (see Fig. S-1). Post hoc t-tests revealed that there were prime compatibility effects in both the small PCE group [t(121) = -9.47; p < .001; incompatible = 96.47 % ± .30 vs compatible = 98.79 % ± .14] and the large PCE group [t(120) = -12.03; p < .001; incompatible = 94.07 % ± .47 vs compatible = 99.40 % ± .07]. Likewise, groups differed in accuracy in both compatible trials [t(241) = -3.75; p < .001] and incompatible trials [t(241) = 4.33; p < .001]. However, accuracy differences (compatible minus incompatible) were more pronounced in the large PCE group (5.38% ± .44) than in the small PCE group (2.32 % ± .24) [t(186.37) = -5.99; p < .001]. Lastly, this interaction show a positive correlation between PCE size and priming effect [r = .449, p < .001]. Furthermore, there was an interaction of prime compatibility x flanker congruency [F(1,241) = 71.85, p < .001, η_p^2= .230]. Post hoc t-tests revealed significant differences for all possible contrasts (all p < .001). However, the prime compatibility effect (i.e. compatible - incompatible) was larger in trials with incongruent flankers (4.84% ± .33) than in trials with congruent flankers (2.84% ± .24) [t(242) = 14.349; p < .001]. Likewise, the flanker congruency effect (i.e. congruent – incongruent) was larger in incompatible primes (2.64% ± .24) than in compatible primes (.64% ± .09) [t(242) = 8.469; p < .001]. The reason for this finding is that the accuracy was much lower in prime-incompatible flanker-incongruent trials (93.92% ± .36), than in prime-incompatible flanker-congruent trials (96.57% ± .26), prime-compatible flanker-incongruent trials (98.77% ± .12), and prime-compatible flanker-congruent trials (99.42% ± .06). While all of those differed significantly, the last three were much closer together, which is also in line with previous findings in healthy young subjects (Stock et al., 2016). All other main effects and interactions of the accuracy analyses were not significant (all F ≤ 1.19; p ≥ .275).

Behavioral data: Hit RTs

For the hit RTs, there was a main effect of prime compatibility [F(1,241) = 1494.61, p < .001, η_p^2= .861] with a faster RTs in compatible (410.2 ms ± 2.42) than in incompatible (449.1 ms ± 2.33) trials. There was also a significant main effect of flanker congruency [F(1,241) = 665.66, p < .001, η_p^2 = .734] with faster RTs in congruent trials (420.6 + 2.36) than in incongruent trials (438.7 ms ± 2.34). Significant interactions were found for prime compatibility x PCE group [F(1,241) = 3661.94, p < .001, η_p^2= .600], flanker congruency x PCE group [F(1,241) = 17.12, p < .001, η_p^2= .066], and for prime compatibility x flanker congruency x PCE group [F(1,241) = 5.85, p = .016, η_p^2 = .024] (see Fig. 2). To analyze the latter, we conducted separate analyses for prime compatible trials and prime incompatible trials. In prime compatible trials, there was an interaction of flanker x PCE group [F(1,241) = 19.26, p < .001, η_p^2= .074]: Post hoc testing showed congruency effects in both the small PCE

group [t(121) = 14.63; p < .001; incongruent = 439.0 ms ± 3.51 vs congruent = 416.6 ms ± 3.57] and large PCE group [t(120) = 11.07; p < .001; incongruent = 399.4 ms ± 3.56 vs congruent = 385.7 ms ± 3.36]. When separately compared, groups differed in both congruent trials [t(241) = 6.29; p < .001; small PCE group = 416.6 ms ± 3.57 vs large PCE group = 385.7 ms ± 3.36] and incongruent trials [t(241) = 7.91; p < .001; small PCE group = 439.0 ms ± 3.51 vs large PCE group = 399.4 ms ± 3.56]. However, reaction time differences (incompatible minus compatible) were more pronounced in the low PCE group (22.4 ms ± 1.53) than in the large PCE group (13.7 ms ± 1.24) [t(231.45) = 4.39; p < .001]. In prime incompatible trials, there was no such interaction of flanker congruency x PCE group [F(1,241) = 3.10, p = .079, η_p^2= .013]. Lastly, for the interaction of flanker congruency x PCE size, there was a negative correlation between PCE size and flanker effect [r = -.272, p < .001]. All other main effects and interactions of the hit RT analyses were not significant (all F ≤ .003; p ≥ .953).

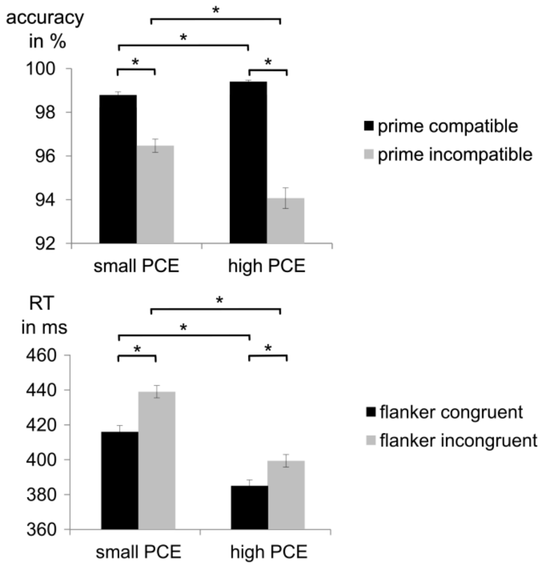

Figure S1
Behavioral Data. There was a positive compatibility effect (PCE) for both accuracy and hit RTs. Accuracy showed an interaction of prime compatibility and PCE group while RTs showed an interaction of prime compatibility x flanker congruency x PCE group. The interaction of prime compatibility and PCE group for both accuracy and RT showed a larger priming effect / difference in the large PCE group as compared to the small PCE group. Significant results (p ≤ .05) are denoted with one asterisk

Speed-accuracy ratio
An interaction of prime compatibility x flanker congruency was obtained [F(1,241) = 11.04, p = 0.001, η_p^2= 0.044]. Post hoc t-tests revealed significant differences for all possible contrasts (all p < 0.001). Yet, the PCE (i.e. compatible - incompatible) was larger in trials with incongruent flankers (0.032 ± 0.001) than in trials with congruent flankers (0.029 ± 0.001) [t(242) = -3.30; p = .001]. Likewise, the flanker congruency effect (i.e. congruent – incongruent) was larger in incompatible primes (0.015 ± 0.001) than in compatible primes (0.012 ± .001) [t(242) = -3.30; p = 0.001]. This was further underpinned by additional t-tests showing that the performance reduction caused by just one kind of conflict (i.e., the induction of either flanker congruency or prime compatibility) was significantly smaller than the performance reduction caused by adding the respective other kind of conflict [all t(242) ≥ |3.302|; p = 0.001].

Neurophysiological data: Prime P1 and prime N1
For the amplitudes of the prime-elicited P1 (pooled across electrodes P7 and P8), there was a significant interaction of prime compatibility x flanker congruency x PCE group [F(1,241) = 4.72, p = .031, η_p^2 = .019]. Yet, this effect did not survive post-hoc testing (all p ≥ .05). All other main effects and interactions were not significant for prime P1 amplitudes (all *F* ≤ 3.01; p ≥ .084).

Neurophysiological data: Target N1

There was an interaction of prime compatibility x flanker congruency [F(1,241) = 12.23, p = .001, η_p^2 = .048]. Post hoc t-tests revealed significant differences for all possible contrasts (all p < .001). Yet, the PCE (i.e. compatible - incompatible) was smaller in trials with incongruent flankers (2.28 µV/m² ± .40) than in trials with congruent flankers (3.71 µV/m² ± .38) [t(242) = 3.50; p = .001]. Likewise, the flanker congruency effect (i.e. congruent – incongruent) was smaller in incompatible primes (2.21 µV/m² ± .37) than in compatible primes (3.063 µV/m² ± .36) [t(242) = 3.50; p = .001].

Neurophysiological data: N2 and P3

Lastly, there was an interaction of prime compatibility x flanker congruency [F(1,241) = 9.17, p = .003, η_p^2 = .037]. Post hoc t-tests revealed significant differences for all possible contrasts (all p < .001). However, the PCE (i.e. incompatible - compatible) was larger in trials with incongruent flankers (4.42 µV/m² ± .41) than in trials with congruent flankers (2.93 µV/m² ± .39) [t(242) = 3.01; p = .003]. Likewise, the flanker congruency effect (i.e. congruent – incongruent) was larger in incompatible primes (4.83 ± .42) than in compatible primes (3.34 µV/m² ± .37) [t(242) = 3.06; p = .003]. The reason for this is that the N2 amplitude was much larger in prime-incompatible flanker-incongruent trials (-17.27 µV/m² ± .97), than in prime-incompatible flanker-congruent trials (-12.44 µV/m² ± .90), prime-compatible flanker-incongruent trials (-12.84 µV/m² ± .91), and prime-compatible flanker-congruent trials (-9.50 µV/m² ± .88). While all of those differed significantly, the last three were much closer together. This was further underpinned by additional t-tests showing that the amplitude increase caused by just one kind of conflict (i.e., the induction of either flanker congruency or prime compatibility) was significantly smaller than the amplitude increase caused by adding the respective other kind of conflict [all t(242) ≥ |3.016|; p = .003]. The type of conflict (i.e., flanker or prime) did not cause significant differences in amplitude changes [all t(242) ≥ |1.052|; p = .294].

For the parietal P3 amplitude (pooled across electrodes PO1 and PO2) (see Fig. S-2), none of the investigated factors or interactions reached significance (all F ≤ 1.04, p ≥ .307).

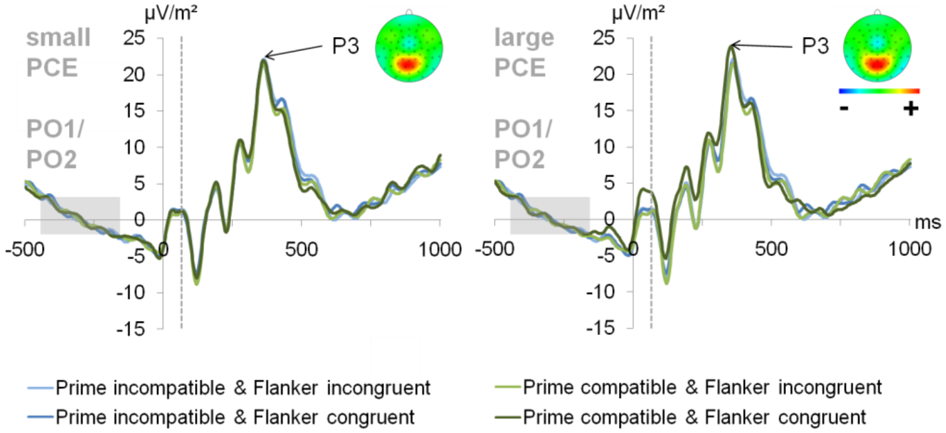

Figure S2

Patietal P3 ERP. The two graphs separately depict the P3 peak for each group (small vs. large PCE effect) at electrodes PO1 and PO2 (pooled). The dashed vertical line in the plot shows the target onset. For each group (small vs large PCE effect), each combination of prime compatibility and flanker congruency is depicted separately (light blue for incompatible primes and incongruent flankers, dark blue for incompatible primes and congruent flankers, light green for compatible primes and incongruent flankers, dark green for compatible primes and congruent flankers). Topography maps of the peaks are depicted right next to the respective peak names. Please note that amplitudes are given in µV/m² due to the CSD interpolation (refer methods section).

Discussion of the interaction between prime and flanker

In line with previous studies (e.g. Gohil et al., 2017; Stock et al., 2016), we found behavioral performance decreases to be potentiated (i.e. disproportionately increased) when both kinds of conflict (i.e. incompatible primes and incongruent flankers) were combined. Hence, it can be stated that the combination of subliminal and conscious conflicts produced a stable conjoint modulation (Gohil et al., 2017; Stock et al., 2016). This supports the assumption that cognitive control may share common processes with unconscious brain mechanisms (Boy et al., 2010), and that the distinction between conscious and non-conscious executive processing might be less clear than originally thought, as awareness does not seem to be an indispensable prerequisite(Huber-Huber and Ansorge, 2018). With respect to the underlying cognitive sub-processes, we found that the two conflicts interact at the N2 amplitude. Importantly, this interaction parallels the pattern observed in the behavioral data, as they N2 amplitudes increase in a non-additive way when the respective other kind of conflict is also present. This increase of N2 amplitudes in case of conflicts is a typical finding (Larson et al., 2014) and is generally thought to reflect an increase in response selection-related conflict and cognitive effort (Botvinick et al., 2004; Folstein and Van Petten, 2007; Larson et al., 2014). We were hence able to reproduce the finding that subliminal conflict load (i.e. a response conflict due to an incompatible prime) may negatively affect consciously perceived response conflicts (e.g. Gohil et al., 2017; Stock et al., 2016).

References

Botvinick, M. M., Cohen, J. D., and Carter, C. S. (2004). Conflict monitoring and anterior cingulate cortex: an update. *Trends in Cognitive Sciences* 8, 539–546. doi:10.1016/j.tics.2004.10.003.

Boy, F., Husain, M., and Sumner, P. (2010). Unconscious inhibition separates two forms of cognitive control. *Proceedings of the National Academy of Sciences* 107, 11134–11139. doi:10.1073/pnas.1001925107.

Folstein, J. R., and Van Petten, C. (2007). Influence of cognitive control and mismatch on the N2 component of the ERP: A review. *Psychophysiology* 0, 070915195953001-??? doi:10.1111/j.1469-8986.2007.00602.x.

Gohil, K., Bluschke, A., Roessner, V., Stock, A.-K., and Beste, C. (2017). ADHD patients fail to maintain task goals in face of subliminally and consciously induced cognitive conflicts. *Psychological Medicine*, 1–13. doi:10.1017/S0033291717000216.

Huber-Huber, C., and Ansorge, U. (2018). Unconscious conflict adaptation without feature-repetitions and response time carry-over. *J Exp Psychol Hum Percept Perform* 44, 169–175. doi:10.1037/xhp0000450.

Larson, M. J., Clayson, P. E., and Clawson, A. (2014). Making sense of all the conflict: A theoretical review and critique of conflict-related ERPs. *International Journal of Psychophysiology* 93, 283–297. doi:10.1016/j.ijpsycho.2014.06.007.

Stock, A.-K., Friedrich, J., and Beste, C. (2016). Subliminally and consciously induced cognitive conflicts interact at several processing levels. *Cortex* 85, 75–89. doi:10.1016/j.cortex.2016.09.027.
